# Addressing Biomechanical Errors in the Back Squat for Older Adults: A Clinical Perspective for Maintaining Neutral Spine and Knee Alignment

**DOI:** 10.3390/jfmk9040224

**Published:** 2024-11-07

**Authors:** Zacharias Papadakis, Andreas Stamatis, Rania Almajid, Kwadwo Appiah-Kubi, Matthew Lee Smith, Nata Parnes, Ali Boolani

**Affiliations:** 1 Human Performance Laboratory, Department of Health Promotion and Clinical Practice, College of Health and Wellness, Barry University, Miami Shores, FL 33161, USA; 2 Health and Sport Sciences, University of Louisville, Louisvile, KY 40292, USA; coach_stam@rocketmail.com; 3 Sports Medicine Institute, University of Louisville Health, Louisvile, KY 40202, USA; 4 Department of Physical Therapy, School of Health Sciences, Stockton University, Calloway, NJ 08205, USA; rania.almajid@stockton.edu; 5 Department of Physical Therapy, Clarkson University, Potsdam, NY 13676, USA; kappiahk@clarkson.edu; 6 Department of Health Behavior, Center for Community Health and Aging, School of Public Health, Texas A&M University, College Station, TX 77843, USA; matthew.smith@tamu.edu; 7 Carthage Area Hospital, Carthage, NY 13619, USA; nparnes@cahny.org; 8 Human Performance and Nutrition Research Institute, Oklahoma State University, Stillwater, OK 74078, USA; ali.boolani@okstate.edu; 9 Department of Physiology and Pharmacology, College of Health Sciences, Oklahoma State University, Stillwater, OK 74107, USA

**Keywords:** hyperlordosis, knee valgus, elderly, biomechanical deficits, quality of life

## Abstract

**Background/Objectives:** Falls pose a significant health risk for older adults, often due to balance disorders and decreased mobility. **Methods:** The ability to perform sit-to-stand transfers, which involve squatting, is crucial for daily independence. Incorporating squats into exercise routines can enhance lower body strength, reduce fall risk, and improve overall quality of life. **Results:** While the back squat is beneficial, proper form is essential to avoid biomechanical errors, like lumbar hyperlordosis and knee valgus. **Conclusions:** Health and fitness professionals, such as physical therapists and/or clinical exercise physiologists, should carefully guide older adults in performing the back squat, addressing any functional deficits, and ensuring proper technique to minimize the risk of injury and maximize the benefits.

## 1. Introduction

The U.S. elderly population (age 65+) is rapidly growing, comprising 17.3% of the nation’s population in 2022. By 2040, it is projected to reach over 80.8 million, with baby boomers accounting for 22% of the total U.S. population by 2040 and 94.7 million by 2060 [1,2]. Older adults face complex health challenges, including age-related chronic conditions and falls. Falls can lead to serious health problems, long-term disability, and significant healthcare costs [3,4]. In the U.S., approximately 30% of older adults experience falls annually. This translates to over 14 million individuals, or 1 in 4, reporting a fall each year [5]. Fall-related deaths are rising dramatically, and healthcare costs associated with these incidents exceed USD 50 billion annually. As the population ages and faces comorbidities, falls are becoming a pressing public health issue [6].

Leading causes of falls in older adults include balance disorders and decreased mobility, often associated with lower quality of life, functional dependence, institutionalization, and mortality [7,8]. The ability to independently perform sit-to-stand (STS) transfers is essential for older adults’ daily safety. Many falls occur during everyday activities like rising from a chair [9,10]. Older adults perform an average of 45 chair stands daily, with chair transfers involving squatting ranging from 33 to 71 STSs [10,11].

Functional assessments, like the 30-Second Sit-to-Stand Test (30s-STS), are crucial in geriatric rehabilitation. They evaluate functional lower extremity strength and are widely used in clinical settings [12]. Squatting is a key component of the 30s-STS, directly assessing functional lower body muscle strength and endurance, including the quadriceps, hamstrings, and glutes [13,14]. Successful sitting-to-standing movement and squatting require good biomechanical strength in the knee extensor muscles and a high level of recruitment from the trunk muscle to provide stabilization for the spine and torso [15].

Incorporating squats into a regular exercise routine can enhance lower body strength, making it easier for older adults to perform daily activities like transfers independently and safely. This can improve quality of life and reduce the risk of falls by decreasing the relative force required for these activities [15,16,17]. A home-based bodyweight squat program involving community-dwelling older adults 70 years and older (100 repetitions daily) for four months effectively enhanced neuromuscular adaptations [18], while a separate study revealed that a brief daily exercise routine consisting of 30 s of bodyweight squats led to an increased maximal squat performance in the older adults (aged 60 and above) over a 24-week period [19]. These findings suggest that even short durations of squat exercises can potentially enhance functional fitness in older individuals. Furthermore, programs like these were found to be practical, well-received, and effective in improving performance in various daily activities [20,21,22]. Further, evidence exists that increased lean mass, which may be a consequence of squat training, reduces the risk of fall-related fractures [23,24]. The back squat is often used to improve lower extremity performance, as it closely mimics functional daily activities, like rising from a chair. Due to its multi-segmental execution, maintaining proper trunk posture and technique is crucial. Variations in squat execution can affect biomechanics, influencing muscular demands, joint loading, and the potential for misinterpretation in clinical settings. Additionally, there is a risk of increased injury [14,15]. The evidence suggests that exercises to reduce fall-related injuries must be individualized and supervised [25,26]. Therefore, health and fitness professionals should carefully consider how to progressively incorporate the back squat into geriatric exercise regimens while minimizing associated biomechanical errors to ensure proper form and technique [14,15].

Two common biomechanical errors in the squat technique are lumbar hyperlordosis and knee valgus, especially in older adults. While precise prevalence rates may vary, studies suggest that a significant portion of older adults exhibit these issues. Factors such as age, physical activity level, and underlying health conditions can influence the occurrence of these problems [27,28]. Lumbar hyperlordosis involves excessive arching of the lower back, which can strain the intervertebral discs and facet joints. Knee valgus occurs when the knees angle inward, increasing stress on the knee joint and potentially contributing to osteoarthritis [29]. Using the back squat as a point of reference, this clinical perspective paper aims to guide health and fitness professionals, such as physical therapists and clinical exercise physiologists, in incorporating the back squat into older individuals’ exercise regimens. It highlights key factors to consider, including recognizing excessive lumbar hyperlordosis and knee valgus, two common biomechanical errors associated with the squat.

## 2. The Back Squat

The proper back squat technique is essential for maximizing performance and minimizing injury risk. This technique can be broken down into three main phases: the starting position, the descent phase, and the upward recovery phase. In the starting position, the individual stands with the barbell resting across the upper back, near the C7 vertebrae and trapezius muscles. A shoulder-width or slightly wider handgrip is maintained, ensuring comfort and security. It is crucial to align the head, shoulders, hips, knees, and ankles vertically to establish a stable and balanced foundation. During the descent phase, the torso remains rigid as the hips, knees, and ankles flex in a controlled manner. The individual lowers themselves until the thighs are parallel to the ground or slightly below. To maintain proper form, the shins and torso should remain parallel, avoiding any rounding of the thoracic or lumbar spine, bending forward at the waist, or allowing the knees to drift inward. The upward recovery phase begins once the individual reaches the lowest point of the squat. The hips, knees, and ankles extend while maintaining a rigid torso. It is important that the hips and shoulders rise at the same rate until the knees are fully extended. Throughout this phase, the individual should avoid rounding the back, bending forward, raising the heels, or looking down, as these errors can compromise the effectiveness of the squat and increase the risk of injury [14,15,29,30,31].

Incorrect back squat performance may be caused by various factors, such as misunderstanding exercise instructions, poor neuromuscular control, insufficient muscle strength or joint stability, or limited joint mobility [32]. Once potential functional deficits are identified, targeted corrective interventions can be implemented to address them. Health and fitness professionals should begin by assessing if the client has misunderstood the exercise instructions, as this could be the underlying cause of poor back squat performance. Clear, concise, and age-appropriate instructions are essential [29,32]. It is important to accommodate anatomical variations in older individuals and adjust their squat form accordingly. For example, individuals with hip retroversion may benefit from a wider stance, greater toe-out position, and reduced squat depth to compensate for limited hip joint range of motion [33]. For further information on back squat functional deficits and factors, readers are encouraged to consult the seminal papers by Kushner et al., Myer et al., and Ronai and Gendron [14,29,32].

This clinical perspective utilized the Back Squat Assessment (BSA) tool developed by Myer et al. to systematically screen for movement deficits during the back squat. The BSA focuses on two common biomechanical errors: lumbar hyperlordosis and knee valgus. The BSA tool includes ten criteria categorized into three domains: upper body, lower body, and movement mechanics. The upper body domain assesses head, thoracic, and trunk position. The lower body domain evaluates hip position, frontal knee position, tibial progression angle, and foot position. The movement mechanics domain focuses on the descent phase, squat depth, and ascent phase. Each criterion has a defined “normal” movement pattern allowing for a systematic evaluation of potential deficits [32].

## 3. Addressing Biomechanical Errors

### 3.1. Lumbar Spine Hyperextension (“Hyperlordosis”)

During the back squat, the neck should be kept neutral with the eyes facing forward to maintain proper alignment and reduce the risk of cervical strain [32,34,35]. The chest should be held erect with retracted scapulae to promote thoracic extension and stability, which helps in maintaining a neutral spine and preventing excessive lumbar lordosis [32,34,35,36]. A slight lumbar lordosis, representing the natural curve of the lower back, is necessary to maintain a neutral spine position. However, excessive lumbar extension, known as hyperlordosis, can reduce intervertebral and facet joint space (i.e., the close-packed position), leading to increased compressive forces on these structures [34,35,37]. The close-packed position refers to the position of the lumbar vertebrae where both the superior and inferior joint surfaces of the vertebrae and the facet joints are in maximal congruency, resulting in higher impaction forces. This impaction force is accentuated when the spine is subjected to increased activation of the back-extensor muscles, particularly during deeper squats [38]. While increased activation of the back extensors can be beneficial for bone remodeling and density [39,40], maintaining a neutral spine during squats is essential to minimize excessive lumbar lordosis and reduce impaction forces [38]. For older individuals, it is crucial to focus on maintaining a neutral spine and avoiding excessive lumbar lordosis to prevent exacerbating conditions such as degenerative disc disease, osteoarthritis, and spinal stenosis [41]. Incorporating core strengthening and flexibility exercises can help older adults maintain proper spinal alignment and reduce the risk of injury.

To mitigate the risks of lumbar hyperextension, individuals should focus on maintaining a neutral pelvis throughout the squat, often achieved through core bracing and controlled breathing. Additionally, improving hip mobility can help reduce compensatory lumbar extension, allowing for proper depth without excessive spinal stress [42]. Regular coaching cues such as “brace your core” and “keep your ribs down” are essential for maintaining proper spinal alignment and minimizing the risk of hyperlordosis during squatting [14,29,32].

For older individuals, a thorough medical history and back examination should precede squatting activities to ensure proper technique and appropriate exercise modifications [43]. While back-extensor strengthening through squats can improve bone resilience, fitness professionals must monitor for pain or injury exacerbation, particularly in individuals with lumbar spine pathologies.

### 3.2. Initial Intervention Recommendations: Maintaining Neutral Spinal Alignment

**Figure 1:**
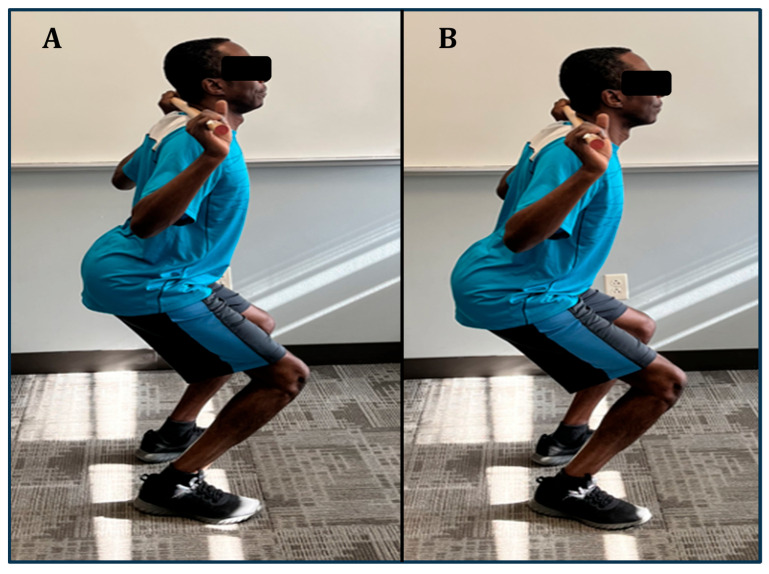
An individual performing back squat with an (**A**) exaggerated lumbar lordosis and (**B**) maintaining neutral spinal alignment. Exaggerated lumbar lordosis (**A**) is associated with hyperextension of both shoulders, which is, in turn, associated with the elbows moving more posteriorly.

The consensus is that proper back squat form requires maintaining a neutral spine and proper hip hinge mechanics to avoid excessive lumbar flexion or extension [14,29,32]. Individuals performing squats may exhibit slight lumbar flexion past the neutral zone with low variability of their lumbar spinal adjustments [31,44]. Excessive lumbar lordosis may aggravate symptoms of spinal stenosis [45] (Figure 1).

Additionally, exaggerated reversal of lumbar lordosis into lumbar flexion may cause vertebral stress fractures in individuals with osteoporosis, a prevalent condition among older adults [46,47]. However, completing a squat with a controlled load while using good body mechanics may potentially increase bone mineral density in clients with osteoporosis [48]. Therefore, when working with older adults, a more individualized intervention approach with very minimal or no pain symptoms in the spine is required.

To address hyperlordosis during the back squat, the health/fitness specialist must first determine whether their client has structural (fixed) or functional (movement-dependent) deficits. From a structural perspective, the angle of lumbar lordosis is determined by the shape of the lumbar vertebrae and the height of the intervertebral disks. A thinner dorsal vertebral wedge leads to a more fixed angle in lordosis, whereas a thinner anterior vertebral wedge reverses the lordosis, leading to a fixed position moving into a more neutral or kyphotic position [49,50]). While maintaining lumbar lordosis is recommended, health and fitness professionals should guide older individuals to perform the back squat while maintaining the most comfortable, symptom-free, neutral spine position.

From a functional perspective, lumbar hyperlordosis and reversal are also directly correlated with the amount of anterior and posterior tilt of the pelvis, respectively [51]. Pelvic tilt relates to a rotational motion of the pelvis in the sagittal plane in relation to a stationary femur [52] and can be in either an anterior or posterior direction. To instruct the individual to find the “pain-free zone”, it may be necessary to start by teaching pelvic control in unloaded positions (e.g., supine progressing to quadruped position).

**Figure 2:**
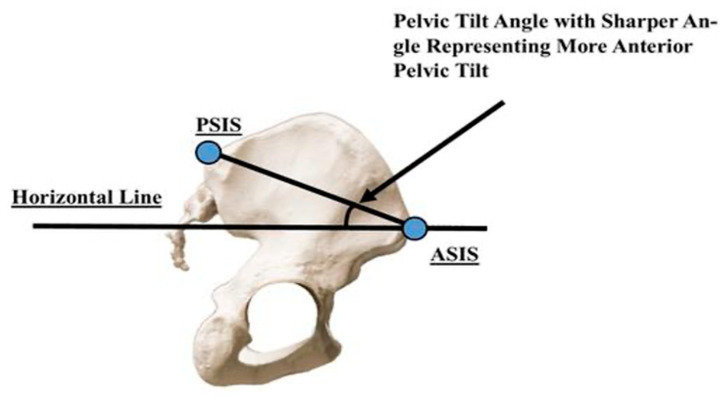
Normal alignment of anterior superior iliac spine (ASIS) and posterior superior iliac spine (PSIS).

Before starting a squat routine with an older individual, the health/fitness specialist must examine the position of the pelvis in a static standing posture. Normal pelvis orientation should be in slight anterior tilt, with an angle between 0 and 5 degrees for males and 7 and 10 degrees for females [53,54]. A way to evaluate the degree and direction of the pelvic tilt is by palpating the anterior superior iliac spine (ASIS) and posterior superior iliac spine (PSIS) on the same side. Normally, the ASIS is horizontally aligned with or slightly below (but no more than 1.5 cm below) the PSIS (Figure 2). Older individuals generally have decreased mobility of the pelvis [55]. It is important for the health/fitness specialist to determine if the client can volitionally change pelvic position and, subsequently, lumbar spine alignment before initiating a squat and, when possible, measure it with valid and reliable measures, such as a caliper-based pelvic inclinometer [54]. For example, if the pelvic tilt in static standing posture is normal but becomes excessive during the squat, the error is more likely functionally derived. In this case, muscle stiffness or tightness may result in imbalanced forces acting on the lumbar spine and pelvis. Functional hyperlordosis can be the result of stiffness or tightness of the anterior hip flexor group, which includes the iliopsoas, tensor fasciae latae (TFL), and rectus femoris, which are most commonly involved [52]. Posteriorly, it is most common to find stiffness and/or tightness in the lumbar extensors, such as the erector spinae and multifidus [52]. A thorough examination of the aforementioned muscles, when deficits are noted, is recommended before progressing into the squat exercise (Table 1).

In addition, some individuals may exhibit motor control deficits, such as poor postural habits, that can lead to biomechanical errors. For example, individuals may exhibit anterior pelvic tilt posture due to poor postural habits and excessive sitting [56]. Therefore, the health/fitness specialist should assess for motor control deficits versus structural deficits and instruct the client to correct that posture [57]. When an individual is able to find and maintain a neutral spine in the unloaded position, the exercise can progress to a loaded position when standing. It is important to recognize if the individual is using hip flexion/extension during a hip hinge motion to complete a loaded or unloaded back squat rather than spinal motions. Spinal flexion/extension should be minimized to prevent injuries, and health/fitness specialists are encouraged to teach clients the bracing technique using the core muscles to maintain neutral spinal alignment in any position [58]. This strategy will enable the client to isolate spinal flexion/extension from hip hinge motion and perform the squat with a hip hinge motion.

**Figure 3:**
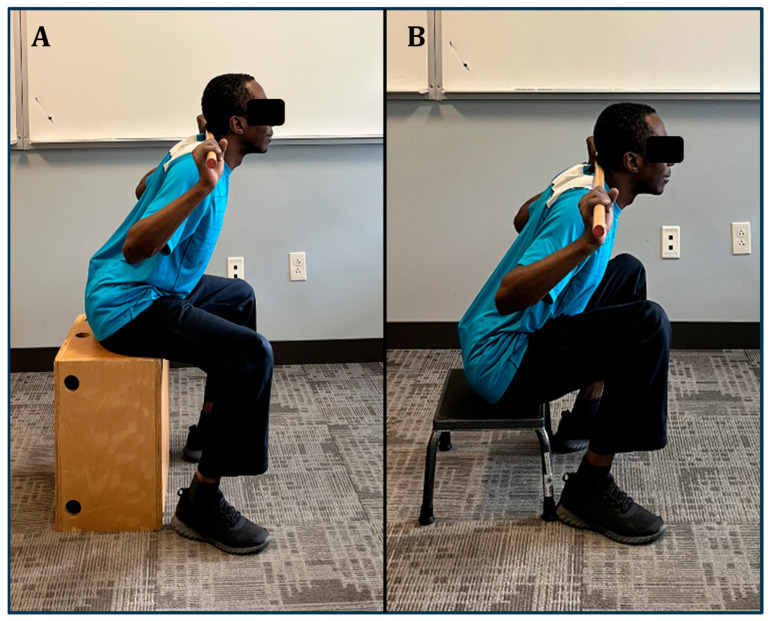
Box squat (**A**) to back squat (**B**) progression.

Once the individual is able to recognize the neutral spine alignment in static standing posture, our recommendation is to start with the box squat exercise as it requires less lower extremity (LE) range of motion (ROM) and center of mass (COM) displacement and lower ground reaction forces [59]. Compared to the back squat, the box squat generates a lower extension peak moment at the L5/S1 level [59]. When the client successfully performs the box squat, the health/fitness expert can progress the older client gradually to a back squat (Figure 3).

The back squat technique can be influenced by the postural changes that occur with advanced aging. Older adults exhibit forward head position, thoracic kyphosis, and decreased shoulder ROM; these changes may also be due to underlying structural or functional deficits [60,61]. Placing a dowel or a bar behind the neck may elicit a compensatory reaction of excessive lumbar lordosis in those with these postural deficits. We suggest starting by replacing the “stiff” dowel with a towel, allowing for a less demanding shoulder ROM. Throughout the entire progression, cueing should emphasize symptom-free, neutral spine, head, and shoulder positions [14,29,32].

### 3.3. Knee Valgus

Another common biomechanical deficit is excessive dynamic knee displacement in the frontal plane during the descent phase of the squat, referred to as “knee valgus” [15,62]. The term “knee valgus” (“knocked knees”) denotes the position of the knee joint in the frontal plane, where the knee is positioned inward, closer to the midline [15,29,32,52]. This position shifts the load at the knee joint to the lateral structures, increasing stress on the lateral collateral ligament, menisci, and patellofemoral joint. Dynamic valgus is often a result of poor neuromuscular control, particularly due to weakness in the hip abductors and external rotators, such as the gluteus medius [63,64].

**Figure 4:**
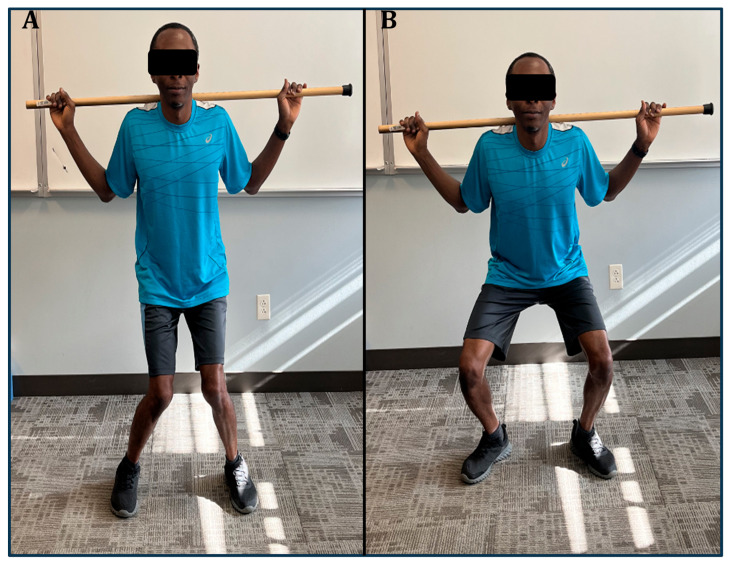
Pathological (**A**) and typical (**B**) valgus angles during squatting.

In static standing, the knee typically maintains a valgus angle of about 6 degrees [37]. A valgus angle exceeding 10 degrees is considered pathological, particularly when accompanied by dynamic movements like squats [65] (Figure 4).

Excessive valgus is a predictor of patellofemoral pain syndrome and ACL injury and may contribute to the progression of knee osteoarthritis, although its direct relationship with osteoarthritis remains complex [66,67]. Older adults, in particular, are at higher risk for knee injuries if valgus collapse is present during squatting, as it may accelerate joint degeneration or contribute to conditions such as chondromalacia or osteochondritis [67,68]. However, it has been reported that the knee joint shift toward relative valgus in older adults shows no radiological osteoarthritic changes [69]. Interventions aimed at reducing knee valgus include strengthening the hip abductors and external rotators, improving core stability, and incorporating neuromuscular training to correct movement patterns [32,70]. Proper coaching cues, such as “knees out”, and the use of resistance bands can further assist in reinforcing correct knee alignment [29,32,64,70,71].

### 3.4. Initial Intervention Recommendations: Knee Alignment

During a back squat, the knee predominantly moves in the sagittal plane, with minimal movement in the frontal plane. Excessive frontal plane movement, such as knee valgus or varus, should be avoided to prevent undue stress on the knee joint [32,70,72]. Knee valgus often results from neuromuscular deficits in the hip abductors and external rotators, as well as restricted ankle dorsiflexion, rather than structural issues [63].

Correcting knee valgus begins with determining whether the alignment deficit is functional or structural. Since the knee is designed for sagittal plane movements controlled by the quadriceps and hamstrings, mediolateral control is largely dependent on the hip abductors, such as the gluteus medius, and external rotators [14,29,32,52,70]. Health and fitness professionals should focus on functional neuromuscular training rather than isolated strengthening, as studies have shown that functional strengthening leads to greater reductions in dynamic knee valgus [29,32,70,73].

A corrective exercise progression may begin with box squats and the use of a resistance band around the knees to activate the hip abductors and improve motor control [14,15,29,32,58,70]. As motor control improves, the individual can reduce the height of the box, progressing to deeper squats and eventually to a goblet squat, which helps maintain good postural alignment and reduces dorsiflexion demands [14,15,29,32,58,70]. The goblet squat is an effective way to transition to more advanced squat variations, such as the back-loaded squat, while ensuring proper knee alignment and movement mechanics [14,15,29,32,58,70].

Dynamic stability of the knee in the frontal plane is achieved mainly through the timed activation of the hip extensors and abductors and the ankle evertors and invertors. These muscles are primarily the gluteus maximus and medius at the hip joint and the tibialis posterior and peroneals at the ankle [14,15,29,32,58,70,74]. Ankle dorsiflexion ROM plays a critical role in maintaining knee alignment during squatting. Normal ankle dorsi flexion among older individuals is 11-12 degrees [75,76]. For example, during the back squat, the required ankle dorsi flexion ROM may reach 30 degrees or more [77]. Ankle muscular strength and ROM significantly decline with advanced aging, which could impact their ability to perform a back squat [78]. Reduced dorsiflexion has been linked to increased knee valgus, as individuals compensate for limited ankle mobility by allowing excessive pronation and medial knee collapse [77,79,80,81]. The use of a heel wedge during squats can temporarily address dorsiflexion limitations, but long-term strategies should focus on improving ankle mobility [82,83]. For older adults with impaired balance, a squat from a starting position using a heel lift provides increased stability required to regulate balance, regardless of the level of experience [84]. Additionally, strengthening the intrinsic foot muscles through short foot exercises can help correct excessive ankle eversion and reduce knee valgus, particularly in individuals with flat feet or a higher body mass index [85,86].

## 4. Conclusions

The back squat, when properly executed, remains a highly beneficial multi-joint exercise for older adults, promoting improvements in strength, balance, and overall functional independence [87,88,89]. Health/fitness professionals, such as physical therapists and clinical exercise physiologists or exercise physiologists, should take a comprehensive approach to movement analysis, incorporating both manual assessments and technology to provide real-time feedback and ensure safe, individualized programming [88,90]. While this exercise mimics many functional movements, special attention must be given to biomechanical deficits, such as knee valgus or hyperlordosis, to prevent injury. Modifications for those with pre-existing conditions, such as osteoporosis, are essential to safely incorporating the back squat into an exercise routine. By prioritizing proper mechanics and ongoing assessments, older adults can maintain engagement in a safe and effective long-term exercise program [15,29,32,87,91].

## Figures and Tables

**Table 1:** Structural and functional biomechanical deficits associated with lumbar hyperlordosis and excessive knee valgus commonly observed during squat exercise.

Segment	Observed Biomechanical Deficit	Plane	Selected Structural Impairments	Selected Functional Impairments
Lumbar Spine	Hyperlordosis	Sagittal	A thinner dorsal vertebral wedge	Isolated or group muscle stiffness and/or tightness of hip flexors, which include iliopsoas, tensor fascia lata (TFL), and rectus femoris
			Reduced height of the intervertebral disks	Isolated or group muscle stiffness and/or tightness of lumbar extensors, which include erector spinea and/or multifidus
	Flat	Sagittal	A thinner anterior vertebral wedge	Long paraspinal muscles and/or long iliopsoas
Pelvis	Anterior Pelvic Tilt	Sagittal		Isolated or group muscle stiffness and/or tightness of hip flexors, which include iliopsoas, tensor fascia lata (TFL), and rectus femoris
				Long external oblique muscle
	Posterior Pelvic Tilt	Sagittal		Short abdominal muscles, long hip flexors, which include iliopsoas, tensor fascia lata (TFL), and rectus femoris
Knee Joint	Genu Valgus	Frontal	Reduced height of the menisci (especially the lateral meniscus)	Knee joint medial collateral ligament tear or overly stretched
				Decreased hip abductor muscle performance, including the gluteus maximus and medius Increased femoral anteversion, internal rotation of tibia
				Decreased ankle inversion muscles, including the tibialis anterior and posterior

## Data Availability

Not applicable.
